# Porous Organic Nanolayers for Coating of Solid-state Devices

**DOI:** 10.1186/1477-3155-9-18

**Published:** 2011-05-14

**Authors:** Sri D Vidyala, Waseem Asghar, Samir M Iqbal

**Affiliations:** 1 Department of Bioengineering, University of Texas at Arlington, Arlington, TX 76010, USA; 2 Nanotechnology Research and Teaching Facility, University of Texas at Arlington, Arlington, TX 76019, USA; 3 Department of Electrical Engineering, University of Texas at Arlington, Arlington, TX 76011, USA; 4 Joint Graduate Studies Committee of Bioengineering Program, University of Texas at Arlington and University of Texas Southwestern Medical Center at Dallas, University of Texas at Arlington, Arlington, TX 76010, USA

## Abstract

**Background:**

Highly hydrophobic surfaces can have very low surface energy and such low surface energy biological interfaces can be obtained using fluorinated coatings on surfaces. Deposition of biocompatible organic films on solid-state surfaces is attained with techniques like plasma polymerization, biomineralization and chemical vapor deposition. All these require special equipment or harsh chemicals. This paper presents a simple vapor-phase approach to directly coat solid-state surfaces with biocompatible films without any harsh chemical or plasma treatment. Hydrophilic and hydrophobic monomers were used for reaction and deposition of nanolayer films. The monomers were characterized and showed a very consistent coating of 3D micropore structures.

**Results:**

The coating showed nano-textured surface morphology which can aid cell growth and provide rich molecular functionalization. The surface properties of the obtained film were regulated by varying monomer concentrations, reaction time and the vacuum pressure in a simple reaction chamber. Films were characterized by contact angle analysis for surface energy and with profilometer to measure the thickness. Fourier Transform Infrared Spectroscopy (FTIR) analysis revealed the chemical composition of the coated films. Variations in the FTIR results with respect to different concentrations of monomers showed the chemical composition of the resulting films.

**Conclusion:**

The presented approach of vapor-phase coating of solid-state structures is important and applicable in many areas of bio-nano interface development. The exposure of coatings to the solutions of different pH showed the stability of the coatings in chemical surroundings. The organic nanocoating of films can be used in bio-implants and many medical devices.

## Background

The interface between biomedical and nanotechnology is an area of intense research. Integration of biomedical micro/nanoelectromechanical systems (BioMEMS/NEMS) and materials offers tremendous potential to tackle medical problems in the areas of diagnostics, therapy, surgical implants and drug delivery [1]. In past few decades, fluorinated coatings have seen many applications in the fields of biochemistry and tissue engineering [2-4]. These coatings are used to attain low surface energy and corrosion resistance properties in nano- and micro-structured devices [5,6]. Organic composite films can be attained by many techniques, e.g. plasma polymerization, biomineralization, chemical vapor deposition (CVD) and self assembled monolayers (SAM) [7-13]. Two important goals of such coatings are biocompatibility and biostability; especially for the surfaces of medical implants. The biocompatibility and biostability can be achieved by modifying the surface characteristics of the substrates. Thus, surface modification of MEMS/NEMS structures has become one of the most important aspects of medically-related devices.

Structural stabilization of the coatings can be achieved from multiple covalent and hydrogen bonds using self organized silane films [14,15]. Fluorinated surfaces have been studied to modify the surface energy, reduce cell adhesion, increase protein adhesion, and also in the development of organic-inorganic hybrid alloys [16-18]. 3-Aminopropyltrimethoxysilane (APTMS) and 1*H*,1*H*,2*H*,2*H*-Perfluorooctyl-trichlorosilane (PFTS) are non-toxic monomers commonly used to create fluorinated surface [7,19]. APTMS, being hydrophilic monomer, is used as a linker in various applications such as for cell adhesion and DNA/protein attachment. It exhibits high coagulation activity [20-23]. APTMS film morphology has been shown to depend on the deposition method [24]. PFTS is highly reactive and being a hydrophobic monomer has relatively low level of coagulation activity due to fluorine rich functional groups [25]. Fluorine groups are inert, homo-compatible and are thermally resistive, which give reduced protein/cell adsorption on surfaces [19,26].

**Figure 1:**
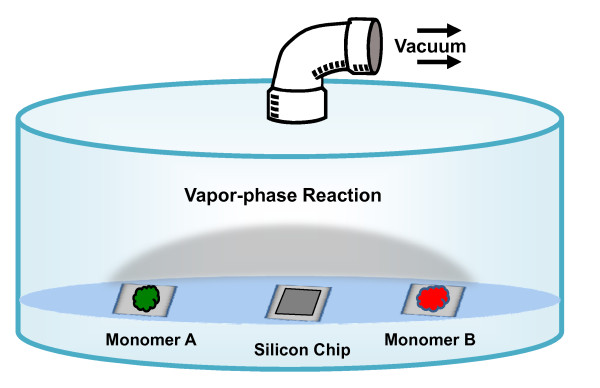
**Schematic illustration of the vapor-phase nanocoating deposition vacuum chamber and monomers (not to scale)**. Monomer A and B depict APTMS and PFTS chemicals placed on two glass slides. The silicon chip to be coated is on middle glass slide. The two monomers react in the vapor-phase and form a gaseous combination above the plain Si chip in vacuum.

This paper reports a novel vapor-phase approach to coat solid-state substrates with a complex of APTMS and PFTS directly by controlling the vacuum (Figure 1). The variations in the chemical composition and stability of the coating with respect to the relative concentrations of the two monomers are studied. Ultrathin nanocoatings were made on the silicon micropore structures using this approach, depositing the monomers atom by atom, but without the need for ultra-high vacuum or harsh chemicals [27-29]. The coating of 3D structures can help in the surface modification of MEMS and nanoscale devices which can be applicable in biochemical/medical areas.

## Results and Discussion

The morphology and the surface chemistry of the nanocoatings are critical factors which determine the biocompatibility and biostability of the films in biomedical applications. Vapor-phase deposition resulted in smooth continuous films. The thicknesses of coatings were measured with respect to the deposition time as shown in Table 1.

**Table 1:** Thickness of the nanolayer with respect to time.

Sample	Deposition Time (mins)	Thickness of the layer formed (nm)
A	20	-
B	30	125.5
C	40	196
D	50	249.6
E	60	312.5

Data is for 2.5:1 ratio of APTMS:PFTS

**Figure 2:**
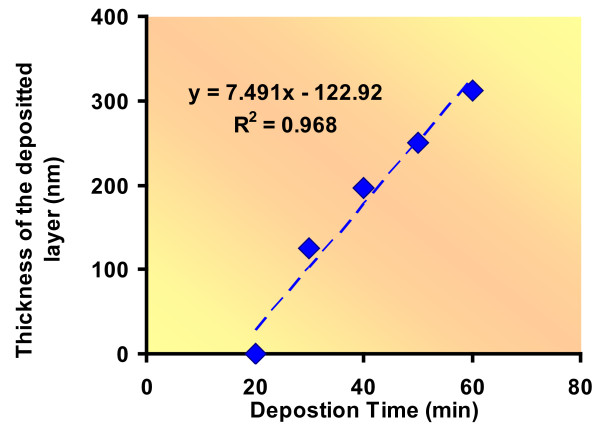
**Thickness of the nanolayer with respect to deposition time**. The thickness of the nanolayer increased as the deposition time increased. The data depicts time of deposition and the respective measured thickness of the layer.

**Figure 3:**
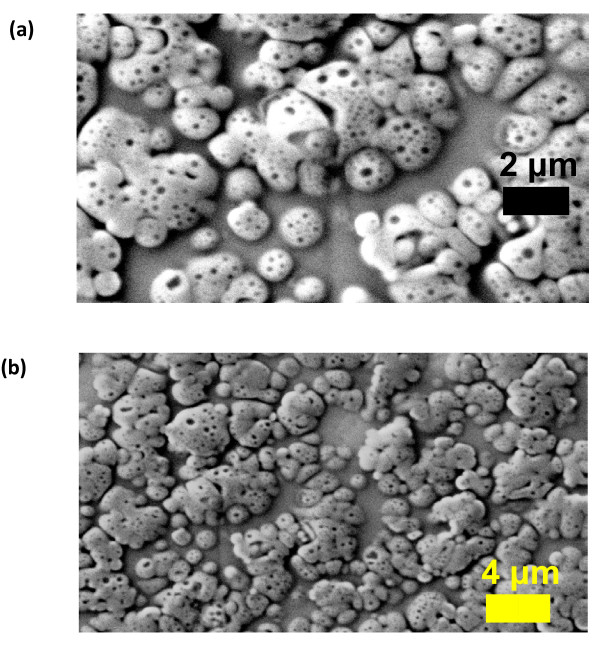
**SEM micrographs of the nanocoating**. SEM micrographs of the nanocoating after 60 minutes of deposition from 2.5:1 of APTMS:PFTS under 22 mmHg at temperature of 40 °C (sample E). The film is 312.5 nm thick with pores in the film ranging in size between 100 - 500 nm. (a) and (b) are at magnifications 23.62 KX and 11.07 KX respectively.

The films formed after 60 minute deposition were thick, continuous and porous at nanoscale (sample E). Figure 2 shows the deposition trend of the nanolayer. The pores on the film were in the range of 100 - 500 nm. Figure 3(a) and 3(b) show the Scanning Electron Microscope (SEM) micrographs of the organic film.

Surface energy was measured with respect to the variations in the relative concentrations of the polymers. An uncoated Si chip was used as control to analyze the difference in the film properties. Table 2 shows the surface energy values with variations in the ratio of APTMS and PFTS. It showed that chemical composition of the surface followed the change in PFTS concentration as the surface energy was least with the concentration ratio of 1:2 between APTMS and PFTS compared to 1:1 and 2:1 concentrations of the two monomers used.

**Table 2:** Calculated Surface Energy

Concentration of APTMS: PFTS	**Average Surface Energy (mJ/m**^**2**^)
Control	62.005
1:2	5.874
2:1	11.336
1:1	10.574

### Spectroscopic Analysis of the Monomers

The chemical compositions of the organic films were analysed using FTIR. Nanolayers were made with 3 different ratios of APTMS and PFTS and their chemical composition was studied.

**Figure 4:**
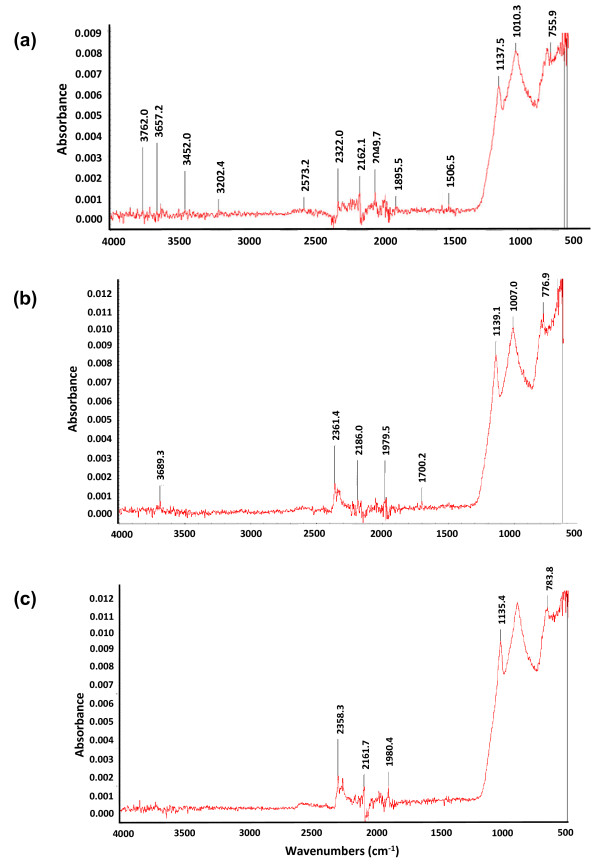
**FTIR spectra of the organic film coated chips with different ratios of APTMS and PFTS**. (a) shows the FTIR spectra for film made from 2:1 ratio of APTMS and PFTS. Broad stretching in the range of 2500 - 3200 cm^-1 ^is observed indicating the presence of O-H and C-H bonds. Small peak at 1700 cm^-1 ^indicates the presence of -C = O bonds. The fingerprint region below 1400 had high absorption peaks at 1260 cm^-1 ^and 1070 cm^-1 ^indicating Si-O-Si (siloxane) bonds. Halogen peaks were in the range of 800-600 cm^-1 ^indicating the presence of fluorine due to PFTS. (b) shows the FTIR spectra for film made from 1:2 ratio of APTMS and PFTS. These results showed peaks in the halogen and fingerprint region with broad stretching for C-H bonds. In this spectrum, most of the bonds formed due to APTMS were dominated by the PFTS halogen bonds. These results show the differences in the chemical composition of the organic nanofilm when opposite concentrations of the polymers were used. (c) shows similar data but the APTMS:PFTS concentration used is 1:1. This shows that PFTS is dominant over APTMS.

The surface state analysis of polymeric surfaces have been reported before using X-ray Photoelectron Spectroscopy [7]. The elemental composition was obtained from high resolution peaks of C, O, F, Si and Cl. The peaks of C and O showed that the films were organic and the peaks formed because of C-F bonds depicted high percentage of F in the film. FTIR data for three concentration combinations (1:1, 2:1 and 1:2 of APTMS and PFTS) are shown in figure 4.

**Figure 5:**
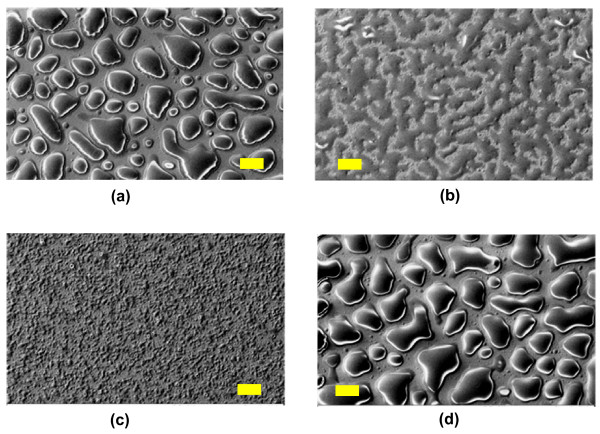
**SEM micrographs of the coated silicon chips dipped in pH solutions**. Coated silicon chips were dipped in solutions with pH 2, 4, 7 and 10 to characterize stability of the nanolayers. (a) shows the coated chip after a 15 hours dip in pH 2 solution. (b), (c) and (d) show the surface of the chips after 15 hours dip in solutions at pH 4, 7 and 10, respectively. All scale bars are 20 μm.

The stability of the nanocoatings was characterized in de-ionized (DI) water and in solutions of various pH values. The surface showed no change when the coated chips were washed with DI water. There was no difference in the contact angle and the surface energy before and after DI water wash of the layer indicating the stability of the layer. But, when the coated chips were left in the DI water for 24 hrs, there was increase in the surface energy. This could be due to hydrolysis of the film in the DI water. The same process was carried out with pH solutions of 2, 4, 7 and 10. The coatings were analyzed from SEM micrographs after these were left immersed in respective solutions for 15 hrs. It was observed that the nanocoatings were still intact on the chips but the stability varied with the pH solution used. Figure 5 shows the micrographs of the nanocoating on chips after dip in various pH solutions. The chip dipped in pH 2 solution showed increased surface energy and very low contact angle. Similar results were seen on the surfaces of chips dipped in pH 10. The pH 4 and pH 7 samples did not show much variation after the exposure to solutions. This indicated that the nanolayer coatings were stable at physiological pH showing biostability and biocompatibility.

### Coating of 3D Structures

**Figure 6:**
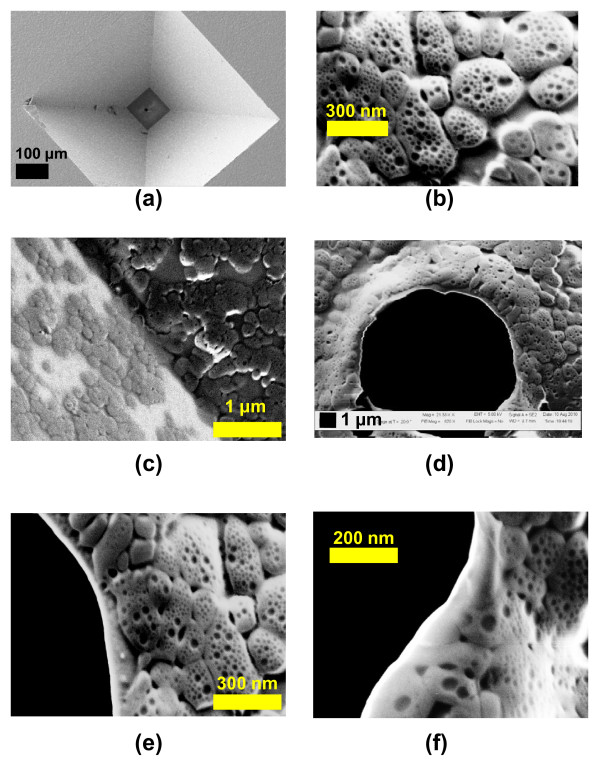
**Coated 3D Solid-state micropore**. The 3D structure of the micropore of radius 11.7 with a nanocoating produced by 2.5:1 ratio of APTMS and PFTS at a vacuum of 22 mmHg for 40 mins deposition. (a) shows the Si substrate with a micropore in it. (b) shows the magnified view of the interconnected coating. (c) Coating on the membrane (darker region) and the inclined wall (bright part). (d) shows the micropore coated with the nanolayer (sample E). (e) and (f) show the periphery of the micropore which shows the inner surface of the pore coated.

The nanolayers were used to coat 3D structures of micro and nanopores. The data showed that micro- or nano-sized structures can be coated evenly on all the sides using this simple approach. Depending on the coating needed, the concentrations of the monomers can be varied. Figure 6(a) shows the SEM micrographs of the 3D surface of a micropore of 11.7 μm diameter. This was coated with 2.5:1 ratio of APTMS and PFTS at a controlled vacuum of 22 mmHg for 40 mins. No change was observed in the pore size as the coating thickness was ~196 nm measured from flat shown in Figure 6(b). Figure 6(c) shows the coating on the angled etched wall of the Si substrate and the membrane of the pore. Figures 6(d) to 6(f) show the SEM micrographs of the coated micropore periphery.

## Conclusion

Nanolayers of biocompatible coatings are the most desired properties for a number of device applications in medicine and engineering. Surface coating of the 3D micro and nano structures are reported using a simple method of vapor-phase vacuum chamber reaction. The coatings show biocompatible, low surface energy fluorinated layers which are ideal for many biomedical applications. The vacuum-based approach helps coating the inner surfaces of the devices and structures without need of any special equipment. This can be helpful in coating medical implants which need to be medicated on all sides of the device. Desired thickness and smoothness of the nanolayers can be acquired with respect to the type of application needed. The characterization showed that the nanolayers are stable at different pH solutions.

## Methods

### Materials Used

APTMS (hydrophilic) and PFTS (hydrophobic) were used as received (Sigma Aldrich). Silicon wafers were <100> orientation p-type doped, oxidized in a thermal oxidation furnace. The wafers were diced into small dyes and used as solid substrates to deposit the nanocoatings.

### Nanolayer Deposition

The substrate was kept in a vacuum reaction chamber and the two monomers were allowed to react in vapor-phase at a controlled vacuum and reaction time allowing the consecutive nanolayer deposition. Schematic diagram of this set up is shown in figure 1. The two monomers were placed on separate glass slides and a glass slide with chip to be coated was placed in between. Vacuum was maintained inside the chamber. The surface morphologies and the smoothness of the film varied with respect to the changes in concentrations of APTMS and PFTS monomers in the reaction chamber [7]. For each concentration combination the film porosity also changed as the film grew thicker.

The thickness of the layer formed was measured with respect to time. The samples were made with different ratios of APTMS and PFTS for 20, 30, 40, 50 and 60 mins of deposition time (Table 1). After the deposition, the vacuum was turned off and the lid of the chamber was kept closed until the pressure meter indicator went down to 0 mmHg.

### Chemical Characterization

The films were made with different concentration ratios of APTMS: PFTS (1:1, 2:1 and 2:1) and the chemical composition of each were characterized using Fourier Transform Infrared Spectroscopy (FTIR). The spectrum was recorded in transmission mode on kBr crystals at a resolution of 4 cm^-1 ^using Nicolet 6700 FTIR spectrophotometer.

### Physical Characterization

The surface energies of the coatings were calculated from the contact angle measurements of the water droplet on the surface of the coated chip [30]. To check the stability of the layer formed, the samples were immersed in the DI water and the surface energy was measured. Different pH solutions (pH 2, pH 4, pH 7 and pH 10) were prepared using HCl and NaOH and the stability of the nanolayer was checked by immersing the coated wafer in these pH solutions for 15 hours.

### Coating of 3D Structures

The 3D micropore structures were coated using these layers. A Si wafer chip with a micropore of size 11.7 μm is shown as an example. The vapor-phase reaction was done using the two monomers. The coating formed a nanolayer on the pore covering all the sides.

## Competing interests

The authors declare that they have no competing interests.

## Authors' contributions

SDV synthesized nanocoatings and carried out the measurements, WA fabricated the micropores, SDV and SMI wrote the manuscript. SMI conceived the design of experiments and supervised all aspects of the work. All authors read and approved the final manuscript.
